# Prevalence of Major Chronic Conditions among Older Chinese Adults: The Study on Global AGEing and Adult Health (SAGE) Wave 1

**DOI:** 10.1371/journal.pone.0074176

**Published:** 2013-09-17

**Authors:** Fan Wu, Yanfei Guo, Paul Kowal, Yong Jiang, Min Yu, Xinjian Li, Yang Zheng, Jiying Xu

**Affiliations:** 1 Shanghai municipal Center for Disease Control and Prevention, Shanghai, China; 2 National Center for Chronic and Non-communicable Disease Control and Prevention, Chinese Center for Disease Control and Prevention, Beijing, China; 3 Department of Health Statistics and Information Systems, World Health Organization, Geneva, Switzerland; 4 Zhejiang Provincial Center for Disease Control and Prevention, Hangzhou, China; 5 Research Centre for Gender Health and Ageing, Faculty of Health, University of Newcastle, Newcastle, New South Wales, Australia; Hunter College, City University of New York (CUNY), CUNY School of Public Health, United States of America

## Abstract

**Background:**

The likely corresponding increase in prevalence of chronic disease will be a major challenge for the health care system. Few nationwide epidemiological studies include a large enough sample of older adults to provide estimates of chronic conditions in the older adult population. This study aimed to estimate the prevalence of eight common chronic health conditions and examine socioeconomic inequalities in the diseases among older adults in China.

**Method:**

Data are from SAGE-China Wave 1, including 13,157 people aged 50-plus years. Respondents were asked if they had been diagnosed with any of the following chronic medical conditions: angina, arthritis, asthma, stroke, diabetes, depression, chronic lung disease and hypertension. A set of validated symptom-based questions and related diagnostic algorithms were also used to estimate disease prevalence for angina, arthritis, asthma and depression. Multivariate logistic regression was performed to examine the probability of developing chronic conditions in relation to sociodemographic variables such as gender, age, urban/rural setting and household wealth level.

**Results:**

Fifty percent of respondents reported having one of the selected chronic conditions, 18.9% two conditions, 5.8% three conditions, and 1.4% reported having four or more chronic conditions. Self-reported prevalence was generated for angina (8%), arthritis (22%), asthma (2%), stroke (3%), diabetes (7%), depression (0.3%), chronic lung disease (8%) and hypertension (27%). The symptom-based prevalence of angina, arthritis, asthma and depression was 10%, 20%, 4% and 2%, respectively.

**Conclusion:**

This study provides the best available prevalence estimates for major chronic health conditions among older Chinese adults. Findings from this study indicated that major chronic conditions were common, so prevention and early intervention targeting adults aged 50 years and older should be prioritized.

## Introduction

China’s population is ageing rapidly, by the end of 2010, the proportion of Chinese aged 60 years and over had reached 13.3% of the total population (Chinese sixth census, 2010). According to demographic forecasts, this proportion is expected to rise to 30 percent by 2030. The absolute numbers are estimated to increase from 128 million in 2000 to 431 million in 2050 [1]. As people age, the risk of chronic conditions increases, such as diabetes, heart disease, cancer and arthritis, with china’s ageing estimated to increase NCD burden by 40% by 2030. Chronic diseases are now among the most common and costly health problems worldwide including China [2], especially among the older population. An estimated 66% of the total Chinese health burden is expected to be in older Chinese adults by 2030 [3]. Given the high rates of modifiable risk factors contributing to the increasing prevalence of chronic disease, more current and detailed information on the epidemiology of chronic conditions, especially among the rapidly growing population of older adults, is urgently needed to anticipate any major challenges for the health care system.

Compared with higher income countries, few nationally representative epidemiological studies with a large sample of older adults are available in lower income countries, in particular, the prevalence of chronic conditions in China. Although several previous large-scale nationally representative studies have reported the prevalence of some chronic conditions, such as CNNHS (China National Nutrition and Health Survey) and Chronic Disease Risk Factor Surveillance in China [4], [5], these studies are not designed specifically for older adults and lack comparability due to differences in the socioeconomic indicators, age groups and the definition of diseases.

The Study on global AGEing and adult health (SAGE) China Wave 1 is a cross-sectional population-based study designed to provide high quality health and well-being data among the older adult population in China. To our knowledge, this was the first nationwide population-based older adults’ health and well-being survey in China. This study uses SAGE-China Wave 1 data to estimate the prevalence of eight common chronic health conditions, examine socioeconomic inequalities in the diseases, and provide information about epidemiologic aspects of chronic conditions among older adults in China.

## Materials and Methods

### Study Population and Design

**Figure 1:**
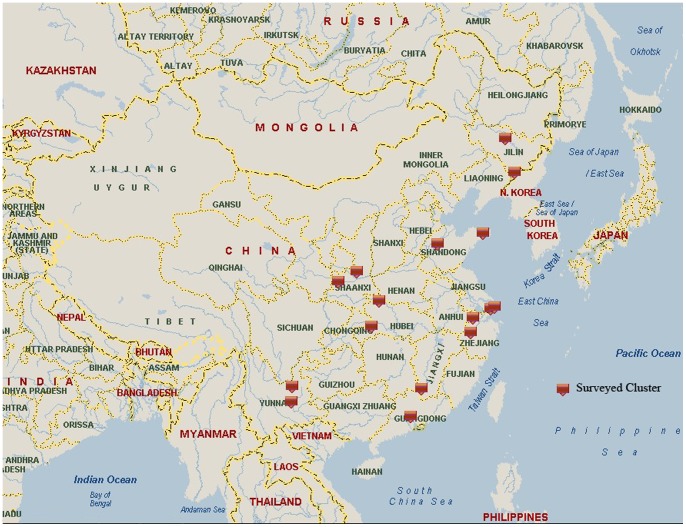
SAGE-China Wave 1 sample distribution.

The World Health Organization’s SAGE is a longitudinal study of ageing and older adults health in six low- and middle-income countries (China, Ghana, India, Mexico, Russian Federation and South Africa) [6]. SAGE-China Wave 1 provides the baseline round of data for a national sample of respondents aged 50-plus. A probability sampling design and a five-stage cluster sampling strategy were used. First, eight provinces/municipalies (Guandong, Hubei, Jilin, Shaanxi, Shandong, Shanghai, Yunnan, and Zhejiang) were selected from a total of 31 provinces/municipalities in China, according to its geographic area and social economic level (Figure 1). Second, one county from rural DSPs (national Death Surveillance Points) and one district from urban DSPs in each province were selected. In total, eight provinces and 16 strata were selected in SAGE-China. The study sample covers a total of 64 principle sample units (PSU) (two urban and two rural townships/communities from each county/district), and 127 secondary sample units (SSU) (2 Villages/enumeration areas (EAs) per township/community) and 254 tertiary sample units (TSU) (2 residential blocks per Village/EA). SAGE-China Wave 1 interviews were completed in 2010, and consists of 1,636 individual respondents aged 18–49 years and 13,177 respondents aged 50-plus years. In this study, the data analysis is restricted to the Chinese adult population aged 50-plus. The response rates for the individual questionnaire was above 98 percent, for a final total sample size of 13,157 for this analysis.

### Chronic Conditions

Information on chronic conditions was based on self-reports. Respondents were asked if they had been diagnosed with any of the following chronic medical conditions: angina, arthritis, asthma, stroke, diabetes, depression, chronic lung disease and hypertension. The question format used was, “Have you ever been diagnosed with/told by a health care professional you have... ?” for each health condition.

In an effort to improve prevalence estimates based on self-report, a set of validated symptom-based questions and related diagnostic algorithms were also used to estimate disease prevalence estimates for angina, arthritis, asthma and depression [7]. In addition, blood pressure was measured three times, at intervals of one minute, to estimate the prevalence of hypertension. An average of the latter two measurements was used as the final blood pressure. The diagnostic criteria and classification of hypertension were based on the Chinese Guidelines on Prevention and Control of Hypertension [8] and the Sixth Joint National Committee on Prevention, Detection, Evaluation, and Treatment of High Blood Pressure guidelines [9]. The definition of hypertension is systolic blood pressure greater than or equal to 140 mmHg and/or diastolic blood pressure greater than or equal to 90 mmHg and/or self-reported treatment of hypertension with antihypertensive medication in the last two weeks.

### Sociodemographic Variables

Socio-demographic variables include age, gender, education, rural/urban residence and income quintiles. For analysis purposes, age is grouped into four categories: 50 to 59 years; 60 to 69 years; 70 to 79 years; and 80-plus years. Education levels were mapped to an international standard and categorized into six groups for analysis [10]. The income quintiles are based on possession of a set of assets and a number of dwelling characteristics [11]. The variable takes on the values Q1 to Q5 with Q1 being the quintile with the poorest households and Q5 the quintile with the richest households.

### Statistical Methods

All analyses were carried out using normalized weights of each individual to compensate for undercoverage, which was based on selection probability, nonresponse, and post-stratification adjustments. These weights are used to calculate self-reported and symptom-based reporting prevalence of chronic conditions. STATA SE version 9 (Stata Corp, College Station, TX) was used in the analyses to calculate prevalence estimates, standard errors, and 95% confidence intervals (CIs). Multivariate logistic regression was performed to examine the relationship between chronic conditions and socio-demographics such as sex, age, urban/rural setting and household wealth. Odds ratios are reported with 95% confidence intervals and a two-side p-value of 0.05 used as the cut-off for statistical significance.

### Ethics Statement

The study protocol was reviewed and approved by the ethical review committee of Chinese Center for Disease Control and Prevention, to ensure the rights and the welfare of the subject are adequately protected; the potential risks are outweighed by potential benefits. Written informed consent was obtained from all participants.

## Results

Sample characteristics were presented in Table 1. From the 13,157 respondents aged 50 -plus years in the whole sample, 6,150 (48.1%) were men and 6,964(51.9%) were women. The overall mean age of was 62.6 years (SD 0.3). The majority of the respondents were between 50 and 59 years old (44.9%), nearly half of all respondents (47.3%) lived in a rural area. Fifty-eight percent had completed primary school or beyond.

**Table 1:** Characteristics of the Study Population, SAGE China Wave 1.

Items	Men		Women		Total	
	No.(weighted)	% (Weighted)	No.(weighted)	% (Weighted)	No.(weighted)	% (Weighted)
**Mean age (SD)**	62.1	0.3	63.03	0.3	62.57	0.3
**Age group**						
50–59	2,633	46.8	3,062	43.1	5,695	44.9
60–69	1,867	32.6	2,052	31.2	3,919	31.9
70–79	1,319	16.7	1,451	20.5	2,770	18.6
80+	348	4.0	425	5.2	773	4.6
**Residence**						
Urban	2,843	43.9	3,576	50.7	6,419	47.3
Rural	3,324	56.1	3,414	49.3	6,738	52.7
**Education**						
No formal education	898	13.1	2,446	33.0	3,344	23.1
Less than primary	1,107	18.4	1,240	19.4	2,347	18.9
Primary school completed	1,434	24.5	1,155	17.6	2,589	21.0
Secondary school completed	1,414	23.4	1,191	16.4	2,605	19.9
High school completed	914	14.6	763	10.7	1,677	12.6
College or Post graduate degree completed	400	6.1	195	3.0	695	4.6
**Income quintile**						
Lowest	1,186	15.8	1,442	16.7	2,628	16.3
Second	1,239	18.4	1,365	17.9	2,604	18.1
Middle	1,268	20.6	1,373	20.4	2,641	20.5
Fourth	1,271	23.5	1,412	23.3	2,683	23.4
Highest	1,186	21.7	1,372	21.8	2,558	21.8

**Figure 2:**
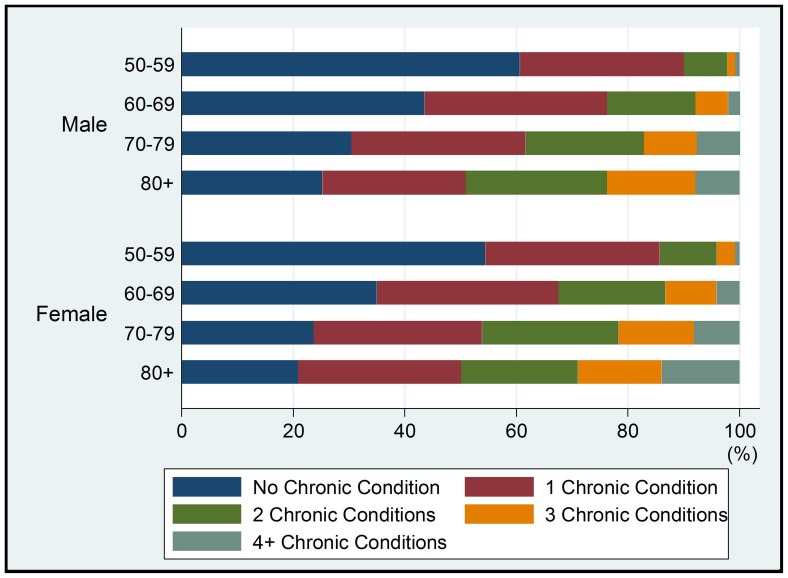
Prevalence of chronic conditions by self-report and symptom reporting.

The self-reported prevalence of the eight major chronic conditions was estimated for the older population (See Table 2). These conditions were commonly reported among respondents, especially among the oldest–old (aged 80-plus years), and varied by condition. Hypertension and arthritis were most commonly reported, with a prevalence of 26.8% and 22.0% respectively, whereas asthma and depression were least common in both sexes, with a prevalence of 2.0% and 0.3%, respectively. Besides self-reported prevalence, symptom-reported prevalence was also examined for angina, arthritis, asthma and depression. Self-reported prevalence of arthritis was close to the symptom-based reporting prevalence (22% vs. 20.4%), whereas the symptom-based reporting prevalence was higher than the self-reported rates for angina (10.0% vs. 7.9%), asthma (3.9% vs. 2.0%) and depression (2.0% vs. 0.3%). The prevalence of hypertension based on measurements, was 59.7%, over two times higher than the self-reported rate (Figure 2).

**Table 2:** Self-reported Prevalence of Major Chronic Conditions Among older adults aged 50 and older in China, by selected sociodemographic characteristics.

	Arthritis		Stroke		Angina		Diabetes		Chronic Lung Disease		asthma		Depression		Hypertension	
	%	95% CI	%	95% CI	%	95% CI	%	95% CI	%	95% CI	%	95% CI	%	95% CI	%	95% CI
**Age group**																
50–59	17.8	[16.1,19.5]	1.5	[1.2,1.9]	4.4	[3.8,5.2]	4.2	[3.6,5.0]	4.7	[4.0,5.5]	1.1	[0.8,1.5]	0.3	[0.2,0.5]	19.0	[17.6,20.5]
60–69	24.8	[22.8,26.9]	3.4	[2.8,4.2]	9.0	[7.8,10.4]	8.0	[6.8,9.4]	8.9	[7.6,10.3]	2.2	[1.8,2.8]	0.3	[0.1,0.6]	29.8	[28.0,31.7]
70–79	26.6	[24.3,29.1]	5.3	[4.4,6.2]	13.1	[10.8,15.9]	9.7	[8.2,11.4]	12.9	[11.1,15.0]	3.5	[2.7,4.5]	0.4	[0.2,1.0]	38.1	[35.8,40.4]
80+	25.5	[22.0,29.5]	7.1	[4.7,10.7]	14.5	[11.2,18.6]	7.7	[5.6,10.5]	13.2	[10.1,17.0]	3	[2.0,4.5]	0.1	[0.0,1.0]	37.3	[32.6,42.4]
**Sex**																
Men	17.6	[15.9,19.5]	3.5	[3.0,4.2]	5.9	[5.1,6.8]	5.7	[4.8,6.9]	9.3	[8.3,10.3]	2.1	[1.7,2.5]	0.3	[0.1,0.5]	23.9	[22.4,25.4]
Women	26.3	[24.5,28.2]	2.6	[2.2,3.1]	9.9	[8.7,11.3]	7.5	[6.4,8.6]	6.6	[5.9,7.4]	1.9	[1.5,2.4]	0.4	[0.2,0.6]	29.7	[28.2,31.2]
**Residence**																
Urban	24.6	[22.3,27.0]	3.7	[3.1,4.5]	10.0	[8.4,11.8]	10.3	[9.2,11.6]	8.9	[7.9,10.0]	2.5	[2.1,3.0]	0.5	[0.3,0.7]	33.8	[31.3,36.3]
Rural	19.6	[17.7,21.8]	2.4	[2.0,2.9]	6.1	[5.3,7.0]	3.2	[2.4,4.2]	7.0	[6.2,8.0]	1.5	[1.1,2.0]	0.2	[0.1,0.3]	20.5	[19.1,21.9]
**Income quintile**																
Q1(Lowest)	21.7	[19.1,24.6]	3.2	[2.5,4.2]	7.1	[5.8,8.7]	2.9	[2.2,3.7]	10.5	[8.9,12.4]	2.8	[2.3,3.5]	0.4	[0.2,0.8]	22.8	[20.4,25.4]
Q2	22.9	[20.6,25.4]	3.1	[2.3,4.2]	8.5	[7.2,10.1]	5.2	[4.2,6.3]	8.7	[7.4,10.2]	1.9	[1.3,2.7]	0.2	[0.1,0.5]	23.3	[21.2,25.5]
Q3	21.9	[19.0,25.2]	3.2	[2.5,4.0]	9.1	[8.1,10.2]	7.1	[5.7,8.7]	7.8	[6.7,9.0]	2.6	[1.9,3.4]	0.3	[0.1,0.9]	26.4	[24.2,28.7]
Q4	21.9	[19.8,24.1]	3.5	[2.8,4.4]	7.5	[6.4,8.8]	7.3	[6.0,8.8]	6.6	[5.4,8.0]	1.3	[1.0,1.6]	0.4	[0.2,0.8]	28.6	[26.5,30.7]
Q5(Highest)	21.7	[18.8,25.0]	2.3	[1.7,3.1]	7.5	[5.3,10.3]	9.3	[7.5,11.4]	6.9	[5.7,8.3]	1.5	[1.0,2.2]	0.2	[0.1,0.6]	30.9	[28.4,33.6]
**Total**	22.0	[20.5,23.6]	3.1	[2.7,3.5]	7.9	[7.1,8.9]	6.6	[5.9,7.4]	7.9	[7.3,8.6]	1.9	[1.7,2.3]	0.3	[0.2,0.4]	26.7	[25.4,28.1]

**Figure 3:**
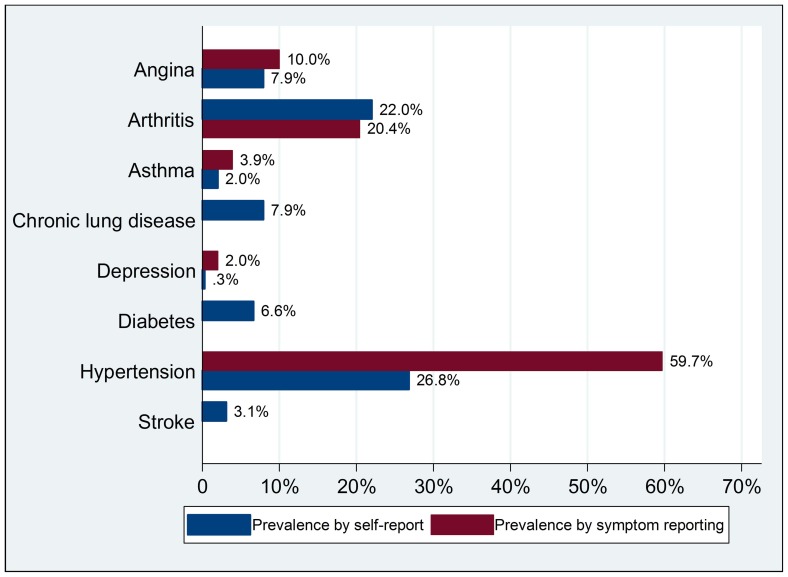
Prevalence of multiple chronic conditions by gender and age group.

As shown in Table 3, comorbid conditions were also common. Based on self-report data, 49.8% of respondents reported one of the selected chronic conditions, 18.9% reported having two conditions, 5.8% reported having three conditions, and 1.4% reported having four or more conditions. Older age, female and urban residence were variables that increased the likelihood of having chronic conditions (Figure 3).

**Table 3:** Prevalence of having no, one and multiple chronic conditions, by age group, sex, residence and income quintile.

	Number of chronic conditions									
	0		1		2		3		≥4	
	%	95% CI	%	95% CI	%	95% CI	%	95% CI	%	95% CI
**Age group**										
50–59	60.1	[57.9,62.2]	29.5	[27.6,31.5]	8.3	[7.4,9.3]	1.6	[1.3,2.1]	0.4	[0.2,0.8]
60–69	45.3	[43.0,47.5]	32.5	[30.8,34.2]	14.9	[13.8,16.0]	5.9	[5.1,6.9]	1.4	[1.0,2.0]
70–79	36.5	[34.5,38.6]	32.6	[30.6,34.8]	19.9	[18.5,21.4]	7.7	[6.3,9.4]	3.3	[2.5,4.3]
80+	40.2	[34.5,46.2]	27.8	[24.0,31.9]	20	[15.7,25.0]	8.7	[6.6,11.3]	3.3	[2.0,5.5]
**Sex**										
Men	54.4	[52.4,56.3]	29.3	[28.0,30.6]	11.6	[10.6,12.6]	3.8	[3.2,4.5]	1.0	[0.7,1.3]
Women	45.9	[43.6,48.3]	32.7	[30.9,34.5]	14.5	[13.6,15.5]	5.1	[4.2,6.1]	1.8	[1.5,2.2]
**Residence**										
Urban	42.3	[39.2,45.4]	32.7	[31.5,33.9]	16.5	[15.1,18.0]	6.2	[4.9,7.7]	2.3	[1.9,2.8]
Rural	57.2	[54.8,59.6]	29.4	[27.6,31.3]	9.9	[9.2,10.6]	2.9	[2.4,3.4]	0.6	[0.4,0.8]
**Income quintile**										
Q1(Lowest)	53.2	[49.6,56.7]	28.9	[26.8,31.1]	12.9	[11.2,14.8]	4.0	[2.9,5.4]	1.1	[0.7,1.6]
Q2	51.7	[49.3,54.2]	30.7	[28.9,32.6]	11.8	[10.4,13.4]	4.3	[3.6,5.3]	1.5	[1.0,2.2]
Q3	50.6	[47.6,53.5]	29.3	[27.0,31.7]	13.9	[12.2,15.8]	4.4	[3.6,5.4]	1.8	[1.3,2.6]
Q4	48.1	[44.7,51.5]	33.8	[31.0,36.7]	13.1	[11.6,14.8]	3.9	[3.2,4.9]	1.1	[0.8,1.7]
Q5(Highest)	48.5	[45.1,51.9]	31.5	[29.7,33.3]	13.2	[11.6,15.1]	5.4	[3.8,7.5]	1.4	[1.0,2.1]
**Total**	50.1	[48.2,52.1]	31.0	[29.9,32.1]	13.0	[12.3,13.8]	4.4	[3.8,5.2]	1.4	[1.1,1.7]

Multivariate logistic regression analyses showed that the prevalence of self-reported chronic conditions differed significantly by sociodemographic variables. Respondents aged 70–79 were more likely to report any chronic condition than those in other age groups. Men were more likely than women to have a stroke, chronic lung disease and asthma. Wealthier respondents were more likely to report diabetes and hypertension. All the selected chronic conditions were more common among urban dwellers, especially diabetes and depression (see Table 4).

**Table 4:** Multivariate logistic regression of the effects of sociodemographic variables on presence of chronic conditions.

	CHRONIC CONDITIONS(Odds Ratio (95% Confidence Interval))							
	Arthritis	Stroke	Angina	Diabetes	Chronic Lung Disease	Asthma	Depression	Hypertension
**Age group**								
50–59	ref	ref	ref	ref	ref	ref	ref	ref
60–69	1.5(1.3–1.7)*	2.3(1.6–3.3)*	2.1(1.7–2.7)*	2.0(1.6–2.4)*	1.9(1.5–2.4)*	1.8(1.3–2.6)*	0.8(0.3–2.1)	1.9(1.6–2.1)*
70–79	1.6(1.4–1.8)*	3.5(2.5–4.8)*	3.0(2.2–4.1)*	2.3(1.9–2.7)*	2.8(2.2–3.6)*	2.7(1.9–4.0)*	1.0(0.3–2.8)	2.6(2.3–2.9)*
80+	1.5(1.2–1.9)*	4.9(2.9–8.4)*	3.5(2.4–5.1)*	2.0(1.3–3.1)*	2.8(2.1–3.9)*	2.3(1.3–4.1)*	0.3(0.0–2.7)	2.7(2.2–3.3)*
**Sex**								
Men	ref	ref	ref	ref	ref	ref	ref	ref
Women	1.6(1.4–1.8)*	0.7(0.5–0.9)*	1.7(1.4–1.9)*	1.2(1.0–1.6)*	0.6(0.6–0.7)*	0.8(0.6–1.1)	1.2(0.6–2.8)	1.3(1.2–1.4)*
**Residence**								
Urban	ref	ref	ref	ref	ref	ref	ref	ref
Rural	0.8(0.7–1.0)	0.7(0.5–0.9)*	0.7(0.5–0.8)*	0.4(0.3–0.5)*	0.7(0.6–0.9)*	0.5(0.4–0.7)*	0.3(0.1–0.6)*	0.6(0.5–0.7)*
**Income quintile**								
Q1(Lowest)	ref	ref	ref	ref	ref	ref	ref	ref
Q2	1.1(0.9–1.4)	1.1(0.7–1.7)	1.4(1.1–1.7)*	1.9(1.4–2.7)*	0.9(0.7–1.1)	0.7(0.5–1.1)	0.5(0.2–1.4)	1.1(1.0–1.3)
Q3	1.0(0.8–1.3)	1.1(0.7–1.5)	1.4(1.1–1.8)*	2.3(1.7–3.3)*	0.8(0.6–0.9)*	0.9(0.6–1.3)	0.6(0.2–2.3)	1.3(1.1–1.5)*
Q4	1.1(0.9–1.3)	1.2(0.8–2.0)	1.2(0.9–1.6)	2.4(1.6–3.6)*	0.7(0.5–0.8)*	0.4(0.3–0.6)*	0.6(0.2–1.7)	1.5(1.3–1.7)*
Q5(Highest)	1.0(0.8–1.2)	0.7(0.5–1.2)	1.1(0.7–1.7)	2.5(1.7–3.6)*	0.7(0.5–0.9)*	0.5(0.3–0.8)*	0.3(0.1–1.3)	1.5(1.2–1.8)*

* Statistically significant at *P*<.05.

## Discussion

SAGE-China Wave 1 is a cross-sectional study which is also part of the multi-country longitudinal WHO SAGE study. In this study, the data analysis is restricted to the Chinese adult population aged 50 years and over. Our study provides the first overview of the magnitude of major chronic conditions based on a nationwide sample of Chinese older adults using self-reported and validated symptom-based reporting methods. Despite several local or national level studies, that have already reported the prevalence rates of one or more chronic diseases in China [4], [12], [13], [14], compared with these studies, the present study has a number of strengths: 1) SAGE China is based on a national probability sample, which can be generalized to Chinese older adults; 2) Validated and standardized questionnaires based on WHO’s World Health Survey to improve cross-national comparability; and, 3) Validated symptom-reporting methods were also used to estimate and compare prevalence rates for several chronic diseases in an effort to improve prevalence estimates based on self-report. However, an important limitation of SAGE is that the data for some chronic diseases were based on self-reports, which may have resulted in recall bias. Self-reported prevalence also may be affected by respondents’ knowledge, their willingness to report the condition, and frequency of contact with a physician.

Findings from SAGE-China Wave 1 show that chronic conditions are common among Chinese older adults. Overall, 50% of Chinese aged 50 years and older reported at least one of the eight major chronic conditions identified, increasing to 78% for those aged 80 years and older. Compared to other available data in China, noting the wide variation in definition of chronic conditions, in the selected survey population, and in sample sizes, the prevalence of multiple chronic conditions among older adults has been reported to range from 50% to 91%. This study generated prevalence-estimates that are generally lower.

Hypertension was most commonly reported among older Chinese men and women, the prevalence of hypertension based on measurement (59.7%) was more than two times higher than self-reported prevalence (26.2%). The prevalence from measured blood pressure was slightly higher than the findings from repeated measurements in the 2002 China National Nutrition and Health Survey (NNHS,2002), where 50% of men and 52% of women aged 60 years or older, had hypertension [15]. The results from another national survey study using repeated blood pressure measurements (chronic disease risk factor surveillance in China, 2007), showed the overall prevalence of hypertension in Chinese aged 60 to 69 years to be 56.9% in both sexes [16], which is similar to the findings of our study. Our results also indicated that less than half of those who were hypertensive were aware of their hypertension status. Dongfeng Gu et,al. [17] also found just 46% of those aged 65–74 with hypertension were aware of their diagnosis. Since hypertension is a leading cause of death and risk factor for stroke, heart disease and diabetes, action should be taken to promote awareness of hypertension in older Chinese adults.

Arthritis is more common among adults, especially older adults [18]. Self-reported arthritis ranked second in this study at 22%, and was very close to the prevalence generated through symptom–reporting method (20.4%). The most likely reason is that the discomfort and pain associated with arthritis are more likely to impact activities and prompt people to seek health care services, as compared to a “silent killer” like hypertension. Angina was ranked as the third most common chronic disease in our study: 7.9% by self-report and 10% using the Rose questionnaire, a recent household-based, community survey showed lower prevalence, 6.5% (95%CI 5.7–7.3) by doctor’s diagnosis and 5.7% (5.0–6.5) using the Rose questionnaire among Chinese 60 years or older [13].

The diabetes rate is increasing in China and becoming a burdensome chronic disease [19], [20]. The present findings for diabetes prevalence were slightly higher than those reported by the 2007 Chronic Disease and Risk Factor Surveillance carried out by China CDC among 49,247 persons aged 15–69 years with an estimated diabetes prevalence for Chinese aged 45–59 and 60–69 to be 3.0% and 5.3%, respectively [16]. Self-reported diabetes prevalence in our study (5.7% in men and 7.5% in women) is much lower than that reported in a previous nationally representative study [21], which found a rate of 20.4% for people 60 years and older using a clinical definition of fasting whole blood glucose of 6.1 mm or higher. This suggests that about half of the respondents are unaware of their diabetic status. Glycosylated haemoglobin results for the SAGE China respondents will be published elsewhere, and could be used to compare to self-report. Stroke represents the third leading cause of death and the second leading cause of disability and dementia in the adult population over 65 worldwide [22]. Self-reported prevalence of stroke in this study was 3.1%. it is very close to the prevalence rate among Chinese Singaporeans aged 50 and older in other study [23]. Also, this present findings for the self-reported prevalence of stroke among older population aged 60–69 (3.4%) is consistent with another study in china that estimated the self-reported prevalence of stroke to be 3.0% among older population aged 60–69 [16].

Depression is one of the most common mental health problems among older adults, but notoriously difficult to assess through health surveys in China [24], [25]. So there has been little information about depression in older Chinese adults. In this study, the self-reported prevalence rate is only 0.3%, and considerably higher using symptom-reporting and diagnostic algorithm (2%), but is still much lower than among western older populations [26], [27]. A meta-analysis on depression of older people in China also found a lower prevalence of depression in mainland China compared to some western countries, and speculated that the possible reasons are Chinese tradition and culture [28]. In addition, as in many other societies, the stigma of mental health is likely present, leading to underdiagnosis and low health care seeking behavior.

In China, respiratory diseases are the third leading cause of death in rural areas and the fourth leading cause of death in urban areas [29]. In SAGE, chronic lung disease is also relatively common among older Chinese men and women. Over 7% of respondents aged 50 years and older reported having chronic lung disease, consisting of 9.3% of men and 6.6% of women. The prevalence of asthma in this study appears to be a little high, compared to prevalence observed in the west, with a rate of 2% by self-report and 3.9% by symptom reporting. However, this finding is very similar to the 2007 China chronic disease and risk factor surveillance with self-reported prevalence of 2.7% among adults aged 53–64 years [16].

In this study, increasing age is associated with higher self-reported prevalence of chronic conditions. It suggests that special attention is needed for older adults in primary care services. Urban/rural inequalities in the prevalence of these chronic conditions were also found in this study, which is a very important finding. All these chronic conditions are more common in urban areas, especially diabetes, hypertension and depression. Other studies also reported higher prevalence of self-reported chronic conditions in urban dwellers [4], [15], [30]. For example, the third Chinese National Health Service Survey (CNHSS), found that the prevalence of self-reported physician diagnoses of chronic diseases was lower among rural than urban residents [10]. The reasons for the high prevalence in urban areas is probably due to (1) the higher rates of common chronic condition risk factors among urban residents compared to rural residents, including low levels of physical activity, air pollution, overweight and obesity; and, (2) persons who live in rural areas being less willing to tell interviewers about their illness due to low education level, traditional understanding of diseases and consuetudinary restriction.

A strong negative income gradient was found in the prevalence of diabetes and hypertension. Even after adjusting for age, sex and urban/rural residence. Wenying Yang et, al. also found diabetes prevalence to be slightly higher in developed region in China as compared to undeveloped regions. Differences in nutrition and lifestyle may play a very important role. Yet SAGE results contrast findings of other studies in developed counties [31], [32]. For example, Rabi, et, al. [31] found that low income is associated with a higher prevalence of diabetes in Canada.

Findings from this study indicated that chronic conditions were common among the older adult population in China. In view of the large older population in China, chronic conditions are likely to be both a health issue and also a social challenge. Prevention and early intervention targeting older adults and urban residents should be prioritized.

## References

[1] United Nations Population Division (2009) World population ageing 2009. New York: United Nations Population Division (UNPD).

[2] World Health Organization (2010) Global status report on noncommunicable diseases.: World Health Organization.

[3] Chatterji S, Kowal P, Mathers C, Naidoo N, Verdes E, et al (2008) The health of aging populations in China and India. Health Aff (Millwood) 27: 1052–1063. 18607041 10.1377/hlthaff.27.4.1052 PMC3645349

[4] Liu S, Wang W, Zhang J, He Y, Yao C, et al (2011) Prevalence of diabetes and impaired fasting glucose in Chinese adults, China National Nutrition and Health Survey, 2002. Prev Chronic Dis 8: A13. 21159225 PMC3044024

[5] Li Y, Zhang M, Jiang Y, Wu F (2012) Co-variations and Clustering of Chronic Disease Behavioral Risk Factors in China: China Chronic Disease and Risk Factor Surveillance, 2007. PLoS ONE 7: e33881. 22439010 10.1371/journal.pone.0033881 PMC3306307

[6] Kowal P, Chatterji S, Naidoo N, Biritwum R, Fan W, et al (2012) Data Resource Profile: The World Health Organization Study on global AGEing and adult health (SAGE). Int J Epidemiol 41: 1639–1649. 23283715 10.1093/ije/dys210 PMC3535754

[7] Moussavi S, Chatterji S, Verdes E, Tandon A, Patel V, et al (2007) Depression, chronic diseases, and decrements in health: results from the World Health Surveys. Lancet 370: 851–858. 17826170 10.1016/S0140-6736(07)61415-9

[8] Committee for Revision of Chinese Guidelines for Prevention and Treatment of Patients with Hypertension (2005) Chinese guidelines for prevention and treatment of patients with hypertension [in Chinese]. Chin J Hypertens 134: 2–41.

[9] National Institutes of Health (1997) The sixth report of the Joint National Committee on prevention, detection, evaluation, and treatment of high blood pressure. Arch Intern Med 157: 2413–2446. 9385294 10.1001/archinte.157.21.2413

[10] United Nations Educational Scientific and Cultural Organization (1997) International Standard Classification of Education (ISCED).

[11] Ferguson B MC, Tandon A, Gakidou E (2003) Estimating permanent income using asset and indicator variables. Health systems performance assessment debates, methods and empiricism. Geneva,Switzerland: World Health Organization. 747–760.

[12] Liu Z, Albanese E, Li S, Huang Y, Ferri CP, et al (2009) Chronic disease prevalence and care among the elderly in urban and rural Beijing, China - a 10/66 Dementia Research Group cross-sectional survey. BMC Public Health 9: 394. 19843346 10.1186/1471-2458-9-394 PMC2770493

[13] Chen R (2012) Prevalence of angina in older adults in China: Performance of the Rose angina questionnaire. Prevention and wellness across the life span. San francisco, CA.

[14] Gu D (2002) Prevalence, Awareness, Treatment, and Control of Hypertension in China. Hypertension 40: 920–927. 12468580 10.1161/01.hyp.0000040263.94619.d5

[15] Wu Y, Huxley R, Li L, Anna V, Xie G, et al (2008) Prevalence, Awareness, Treatment, and Control of Hypertension in China: Data from the China National Nutrition and Health Survey 2002. Circulation 118: 2679–2686. 19106390 10.1161/CIRCULATIONAHA.108.788166

[16] National Center for Chronic and Non-communicable Disease Control and Prevention (2007) Report on Chronic Disease Risk Factor Surveillance in China. BeiJing. 64–71 p.

[17] Gu D, Reynolds K, Wu X, Chen J, Duan X, et al (2002) Prevalence, awareness, treatment, and control of hypertension in china. Hypertension 40: 920–927. 12468580 10.1161/01.hyp.0000040263.94619.d5

[18] Rasch EK, Hirsch R, Paulose-Ram R, Hochberg MC (2003) Prevalence of rheumatoid arthritis in persons 60 years of age and older in the United States: effect of different methods of case classification. Arthritis Rheum 48: 917–926. 12687533 10.1002/art.10897

[19] Pan XR, Yang WY, Li GW, Liu J (1997) Prevalence of diabetes and its risk factors in China, 1994. National Diabetes Prevention and Control Cooperative Group. Diabetes Care 20: 1664–1669. 9353605 10.2337/diacare.20.11.1664

[20] Gu D, Reynolds K, Duan X, Xin X, Chen J, et al (2003) Prevalence of diabetes and impaired fasting glucose in the Chinese adult population: International Collaborative Study of Cardiovascular Disease in Asia (InterASIA). Diabetologia 46: 1190–1198. 12879248 10.1007/s00125-003-1167-8

[21] Yang W, Lu J, Weng J, Jia W, Ji L, et al (2010) Prevalence of diabetes among men and women in China. N Engl J Med 362: 1090–1101. 20335585 10.1056/NEJMoa0908292

[22] Llibre Jde J, Valhuerdi A, Calvo M, Garcia RM, Guerra M, et al (2011) Dementia and other chronic diseases in older adults in Havana and Matanzas: the 10/66 study in Cuba. MEDICC Rev 13: 30–37. 22143605 10.37757/MR2011V13.N4.7

[23] Venketasubramanian N, Tan LCS, Sahadevan S, Chin JJ, Krishnamoorthy ES, et al (2005) Prevalence of Stroke Among Chinese, Malay, and Indian Singaporeans: A Community-Based Tri-Racial Cross-Sectional Survey. Stroke 36: 551–556. 15692124 10.1161/01.STR.0000155687.18818.13

[24] Beekman AT, Deeg DJ, Braam AW, Smit JH, Van Tilburg W (1997) Consequences of major and minor depression in later life: a study of disability, well-being and service utilization. Psychol Med 27: 1397–1409. 9403911 10.1017/s0033291797005734

[25] Bromet E, Andrade LH, Hwang I, Sampson NA, Alonso J, et al (2011) Cross-national epidemiology of DSM-IV major depressive episode. BMC Med 9: 90. 21791035 10.1186/1741-7015-9-90 PMC3163615

[26] Kivela SL, Pahkala K, Laippala P (1988) Prevalence of depression in an elderly population in Finland. Acta Psychiatr Scand 78: 401–413. 3265843 10.1111/j.1600-0447.1988.tb06358.x

[27] McCracken CF, Boneham MA, Copeland JR, Williams KE, Wilson K, et al (1997) Prevalence of dementia and depression among elderly people in black and ethnic minorities. Br J Psychiatry 171: 269–273. 9337983 10.1192/bjp.171.3.269

[28] Chen R, Copeland JR, Wei L (1999) A meta-analysis of epidemiological studies in depression of older people in the People’s Republic of China. Int J Geriatr Psychiatry 14: 821–830. 10521881 10.1002/(sici)1099-1166(199910)14:10<821::aid-gps21>3.0.co;2-0

[29] Lopez AD, Murray CC (1998) The global burden of disease, 1990–2020. Nat Med 4: 1241–1243. 9809543 10.1038/3218

[30] Shi J, Liu M, Zhang Q, Lu M, Quan H (2008) Male and female adult population health status in China: a cross-sectional national survey. BMC Public Health 8: 277. 18681978 10.1186/1471-2458-8-277 PMC2529296

[31] Rabi DM, Edwards AL, Southern DA, Svenson LW, Sargious PM, et al (2006) Association of socio-economic status with diabetes prevalence and utilization of diabetes care services. BMC Health Serv Res 6: 124. 17018153 10.1186/1472-6963-6-124 PMC1618393

[32] Imkampe AK, Gulliford MC (2011) Increasing socio-economic inequality in type 2 diabetes prevalence–repeated cross-sectional surveys in England 1994–2006. Eur J Public Health 21: 484–490. 20685812 10.1093/eurpub/ckq106

